# Peripheral T-Cell Lymphomas Involving the Central Nervous System: A Report From the Czech Lymphoma Study Group Registry

**DOI:** 10.3389/fonc.2022.874462

**Published:** 2022-05-12

**Authors:** Heidi Mocikova, Robert Pytlík, Katerina Benesova, Andrea Janikova, Juraj Duras, Alice Sykorova, Katerina Steinerova, Vit Prochazka, Vit Campr, David Belada, Marek Trneny

**Affiliations:** ^1^Department of Internal Medicine - Hematology, Third Faculty of Medicine, Charles University, Prague, Czechia; ^2^Institute of Haematology and Blood Transfusion, Prague, Czechia; ^3^1st Department of Medicine, First Faculty of Medicine, Charles University, Prague, Czechia; ^4^Department of Internal Medicine, Hematology and Oncology, University Hospital Brno, Brno, Czechia; ^5^Department of Hemato-Oncology, University Hospital and Faculty of Medicine, Ostrava, Czechia; ^6^4th Department of Internal Medicine - Hematology, University Hospital and Faculty of Medicine, Hradec Kralove, Czechia; ^7^Department of Clinical Hematology, University Hospital, Pilsen, Czechia; ^8^Department of Haemato-Oncology, Faculty of Medicine and Dentistry, Palacky University, Olomouc, Czechia; ^9^Institute of Pathology and Molecular Medicine, University Hospital Motol, Prague, Czechia

**Keywords:** peripheral T-cell lymphoma, central nervous system, leptomeningeal infiltration, CNS prophylaxis, methotrexate

## Abstract

**Introduction:**

We analyzed the incidence, risk factors of central nervous system (CNS) relapse, and outcome of CNS involvement in patients with peripheral T-cell lymphomas (PTCL) from the Czech Lymphoma Study Group Registry NiHiL (Clinical Trial gov. NCT03199066).

**Materials and Methods:**

Out of 1,040 patients with PTCL, we identified 29 patients (2.79%) with CNS involvement: 2 patients with primary CNS T cell lymphoma, 11 patients with CNS and systemic disease at diagnosis, and 16 patients (1.54%) at CNS relapse. The most common histology with CNS disease was PTCL, not otherwise specified. Progression-free survival (PFS) was defined as the time interval from diagnosis to progression or death. PFS-2 was defined as the interval from the date of a new relapse until the next relapse.

**Results:**

Patients with testicular involvement received intrathecal prophylaxis with methotrexate. High-dose methotrexate-based treatment was administered in 44.8% of patients with CNS disease. Median follow-up was 71.3 months. The difference between the median PFS of 1,027 patients without initial CNS disease (32.6 months) and 11 patients with initial CNS and systemic disease (4.8 months) was significant (*p* = 0.04). The difference between the median PFS2 in CNS relapses (10.1 months) and 493 relapses outside of CNS (9.1 months) was not significant (*p* = 0.6). Risk factors for CNS relapses included the following: involvement of more than one extranodal site (*p* = 0.008), soft tissue involvement (*p* = 0.003), testicular involvement (*p* = 0.046), and the presence of B symptoms (*p* = 0.035). The difference between the median OS of 1,027 patients without initial CNS disease (46.0 months) and 11 patients with initial CNS and systemic disease (18.2 months) was significant (*p* = 0.02). The median OS2 in CNS relapses was 11.8 months and that in relapses outside of CNS was 21.3 months. CNS involvement was not associated with a significantly worse OS compared to relapsed/refractory patients without CNS involvement (*p* = 0.1).

**Conclusions:**

The incidence of CNS disease at the time of diagnosis and at relapse in PTCL is low and usually associated with other systemic involvement. The prognosis of PTCL with initial CNS involvement is significantly worse when compared to patients without CNS disease at diagnosis. The outcome of CNS relapse is comparable with relapsed PTCL outside of CNS. The optimal treatment is not defined yet.

## Introduction

Central nervous system (CNS) involvement in peripheral T-cell lymphoma (PTCL) is a rare event when comapred with adult T-cell leukemias and HIV-associated T-cell lymphomas. Primary CNS lymphoma exclusively involves CNS at presentation and secondary CNS lymphomas are diagnosed in patients with systemic lymphomas where CNS involvement can occur at presentation or relapse as an isolated site of recurrence or associated with systemic disease (1). Primary CNS T-cell lymphomas (PCNSTCLs) occur in 7.4%–8.5% in Asia and in 3.5%–5% in Western countries (2–5). The brain is the most commonly involved site (93%), followed by the spinal cord (4%) and the meninges (2%) (4). Involvement of deep brain structures (basal ganglia, corpus callosum, brainstem, and/or cerebellum) occurs in 30%–36% of cases (4). The incidence of ocular involvement is low in PCNSTCL (4). Multiple lesions are diagnosed in 29%–56% of patients (4). Secondary CNS T-cell lymphoma at presentation is rare. CNS relapse occurs in various subtypes of PTCL between 2.1% and 8.8% (6–14). PTCL, not otherwise specified (PTCL-NOS) constitutes the majority of PCNSTCL (83%), followed by anaplastic large cell lymphoma (ALCL) (15, 16). PTCL-NOS with gamma-delta origin was reported anecdotally (15, 17, 18). The most common types of histology in secondary T-cell CNS lymphomas are as follows: PTCL NOS, ALK-positive ALCL, and extranodal natural killer/T-cell lymphoma (ENKTCL) (6, 8, 10–12). Secondary CNS dissemination has been reported in 7% of patients with enteropathy-associated T-cell lymphoma (8), and in 10% of patients with monomorphic epitheliotropic intestinal T-cell lymphoma (9). Angioimmunoblastic lymphoma rarely disseminates into CNS.

Based on retrospective studies, several risk factors associated with a higher risk of CNS dissemination have been documented: involvement of >1 extranodal site; involvement of gastrointestinal tract, skin, nose, or paranasal sinuses; advanced disease (Ann Arbor clinical stage III or IV); International Prognostic Index (IPI) score ≥3; and elevated serum lactate dehydrogenase (LDH) (1). A new CNS prognostic index of natural killer lymphoma (CNS-PINK) was developed for patients with ENKTCL treated with non-anthracycline-based chemotherapy (19). A sum of scores was calculated using one-point predictors of CNS relapse (B symptoms, stage III or IV, and distant lymph-node involvement) and extranodal involvement. The low-risk group was defined as 0 or 1 point and the high-risk group of the CNS-PINK model was defined as 2 points. The 2-year rates for CNS relapse were 4.1% for the low-risk group and 22.8% for the high-risk group. The cumulative incidence of CNS relapse was significantly different between the CNS-PINK risk groups in the training cohort (*p* < 0.001) as well as in the validation cohort (*p* = 0.038). Patients in the high-risk CNS-PINK group who received dexamethasone, methotrexate, ifosfamide, L-asparaginase, and etoposide (SMILE) or SMILE-like regimens with intermediate dose of methotrexate (MTX) displayed a lower incidence rate of CNS relapse than did those who received other regimens.

Prognostic indexes to predict CNS involvement in T-cell lymphoma patients other than ENKTCL do not exist. When the CNS-IPI commonly used in aggressive B-cell lymphomas is used in PTCL, the diagnostic sensitivity is only 6% (6). Therefore, it should not be used routinely in patients with T-cell lymphomas (1).

Intrathecal CNS prophylaxis with MTX was not associated with reduced risk of CNS recurrence (8, 10, 11). CNS prophylaxis with intermediate doses of MTX (≥2 g/m^2^) as a part of SMILE or SMILE-like regimens is associated with a significantly lower CNS relapse rate in patients with high-risk ENKTCL (CNS-PINK score of 2), but there was no difference in the low-risk group (19). The role of intermediate or high doses of MTX in combination with regimens containing anthracycline and/or high-dose ifosfamide in enteropathy-associated T-cell lymphoma is difficult to establish (20).

Patients with T-cell lymphomas at high risk of CNS dissemination who receive a high dose of MTX as part of the first-line treatment do not need additional prophylaxis (1). There is a lack of data supporting drugs other than MTX as CNS prophylaxis in T-cell lymphomas.

Treatment of CNS T-cell lymphomas is less effective as compared to primary CNS B-cell lymphomas and no standard approach exists for primary and secondary CNS involvement of T-cell lymphomas. Current treatment options of CNS disease in PTCL are based on the high dose of MTX combined with high doses of AraC and ifosfamide. Immune checkpoint inhibitors are active in patients with ENKTCL who did not respond to L-asparaginase regimens (21). Autologous stem cell transplantation (ASCT) was reported in secondary CNS T-cell lymphomas and followed the recommendation in aggressive B-cell lymphomas (22–26).

## Materials and Methods

### Patients

Overall, 1,326 patients with T-cell lymphomas including 1,040 with PTCL were reported to the Czech Lymphoma Study Group Registry NiHiL between January 1999 and December 2020. All patients signed the written informed consent with participation in the NiHiL data collection and analyses that were approved by the ethics committees in participating centers. Diagnosis of PTCL was confirmed by biopsy and pathology reports from reference hematopathologists. Other types of T-cell lymphomas (adult T-cell leukemia/lymphoma, NK cell leukemia, lymphoblastic lymphoma, ENKTCL, primary cutaneous PTCLs, mycosis fungoides, Sezary syndrome, and HIV-1 positive lymphoma) were excluded from this analysis. Standard workup at diagnosis and at relapse included assessment of ECOG performance status; physical examination; laboratory studies; bone marrow biopsy; computed tomography (CT) of the neck, chest, abdomen, and pelvis; or positron emission tomography(PET)/CT imaging. Patients with neurological symptoms at diagnosis or at relapse underwent neurological and ophthalmologic examinations, magnetic resonance imaging (MRI) of the brain or the spine, and analysis of cerebrospinal fluid (CSF) including cytology and flow cytometry. Patients without neurological symptoms at diagnosis or at relapse were not routinely assessed for CNS disease. Systemic treatment response was assessed by CT or PET/CT and CNS response was evaluated by MRI and CSF examination.

### Statistics

Patient characteristics were compared using the Fisher exact test or Chi-square test. Progression-free survival (PFS) was defined as the time interval from the day of diagnosis until the first objective evidence of relapse/progression or date of the last follow-up or death from any cause. PFS2 was defined as the time interval from the date of a new relapse until the next relapse. Overall survival (OS) was calculated from the date of diagnosis to death from any cause. Survival curves were calculated by Kaplan–Meier survival analysis and a comparison between subgroups was performed by the log-rank test. Risk factor analysis was made by Cox proportional hazards regression. *p*-values < 0.05 were considered statistically significant.

## Results

### CNS Involvement

The characteristics of all patients with PTCL are summarized in **Table 1**. None of these patients were HIV-1 positive. Out of 1,040 patients with PTCL, we identified 29 patients (2.79%) with CNS disease including 13 cases at initial diagnosis (**Table 1**). The characteristics of 16 patients with CNS relapse and 493 patients with systemic relapse are summarized in **Table 2**. The most common histology in CNS disease was PTCL-NOS (**Tables 1**, **2**). The median age at presentation [61.0 (range 29–80) years] and at relapse [54.5 (range 39–75) years] did not differ significantly (*p* = 0.429). There was no statistical difference between male and female patients at initial CNS disease or at relapse (**Tables 1**, **2**). Out of 13 patients, we identified 2 cases with PCNSTCL. CNS disease concurrently with systemic lymphoma involvement at presentation was documented in 11 of 13 patients. Secondary CNS relapses occured in 16 patients (13 at the first relapse, 2 at the second relapse, and 1 at the fourth relapse) and concomitant systemic disease was found in 12 of them (**Table 2**).

**Table 1:** Characteristics of patients with initial CNS disease (*n* = 13) and all peripheral T-cell lymphomas (*n* = 1,040).

Characteristics	Initial CNS disease (*n* = 13 pts)	PTCL without initial CNS involvement (*n* = 1,027 pts)
**Subtype of PTCL**		
PTCL-NOS	10*	406
ALCL ALK neg.	1	157
ALCL ALK pos.	0	74
AITL	1	108
EATL	1	38
MEITL	0	1
Other	0	243
**Age**, median (range) years	61.0 (24–80)	60.0 (18–92)
**Gender,** Male/Female	7/6	600/427
**Clinical stage**		
I(E)	2*	165
II(E)	0	183
III(E)	0	246
IV	11	433
**ECOG PS**		
0	3	329
1	3	381
2	3	200
3	2	90
4	2	27
**B symptoms,** Yes/No		
	7/6	507/520
**Bulky mass ≥10 cm**, Yes/No	2/11	107/920
**Number of extranodal sites**		
0–1	3	819
≥2	10	208
**LDH ≥** ULN, Yes/No	6/7	627/400
**International Prognostic Index**		
0	0	228
1	2	106
2	1	219
3	4	246
4	4	121
5	2	107
**Involved CNS sites**		
Parenchymal	6*	
Leptomeningeal	4	–
Parenchymal and leptomeningeal	2	–
Ocular and leptomeningeal	1	–
		
**Deep brain structures involved**		
Yes/No	3/10	–
**Cerebrospinal fluid protein concentration elevated**		
Yes/No/Missing	6/3/4	–

pts, patients; PTCL, peripheral T-cell lymphoma; NOS, not otherwise specified; ALCL, anaplastic large cell lymphoma; ALK, anaplastic lymphoma kinase; AITL, angioimmunoblastic T-cell lymphoma; EATL, enteropathy-associated T-cell lymphoma; MEITL, monomorphic epitheliotropic intestinal T-cell lymphoma; ECOG PS, the Eastern Cooperative Oncology Group Performance status; LDH, lactate dehydrogenase; ULN, upper limit of normal. *Two patients with primary T CNS lymphoma.

**Table 2:** Characteristics of patients with secondary CNS relapse (*n* = 16) and with systemic relapse of peripheral T-cell lymphomas (*n* = 493).

Characteristics	Secondary CNS relapse (*n* = 16 pts)	PTCL with systemic relapse (*n* = 493 pts)	*p*
**Subtype of PTCL**			
PTCL-NOS	8	224	0.223
ALCL ALK neg.	2	67	
ALCL ALK pos.	2	17	
AITL	1	60	
EATL	1	19	
MEITL	1	0	
Others	1	106	
**Age**, Median (range) years	54.5 (39–75)	61.0 (18.0–91.0)	0.181
**Gender,** Male/Female	7/9	307/186	0.140
**Clinical stage**			
I(E)	1	43	0.04
II(E)	1	85	
III(E)	1	133	
IV	13	232	
**ECOG PS**			
0	3	137	0.835
1	7	195	
2	4	103	
3	2	48	
4	0	10	
**Soft tissue involvement**			
Yes	4	21	0.005
No	12	472	
**Testicular involvement**			
Yes	1	0	0.008
No	15	493	
**B symptoms,** Yes/No	12/4	265/228	0.084
**Bulky mass ≥ 10, cm** Yes/No	1/15	62/431	0.410
**Number of extranodal sites**			
0–1	8	382	0.019
≥2	8	111	
**LDH ≥** ULN, Yes/No	11/5	314/179	0.683
**International Prognostic Index**			
0	0	82	0.039
1	4	48	
2	4	112	
3	2	142	
4	2	55	
5	4	54	
**Involved CNS sites**			
Parenchymal	5	–	–
Leptomeningeal	6	–	–
Parenchymal and leptomeningeal	5	–	–
Ocular and leptomeningeal	0		
**Deep brain structures involved**			
Yes/No	0/16	–	–
**Cerebrospinal fluid protein concentration elevated**			
Yes/No/Missing	1/2/13	–	–

pts, patients; PTCL, peripheral T-cell lymphoma; NOS, not otherwise specified; ALCL, anaplastic large cell lymphoma; ALK, anaplastic lymphoma kinase; AITL, angioimmunoblastic T-cell lymphoma; EATL, enteropathy-associated T-cell lymphoma; MEITL, monomorphic epitheliotropic intestinal T-cell lymphoma; ECOG PS, the Eastern Cooperative Oncology Group Performance status; LDH, lactate dehydrogenase; ULN, upper limit of normal.

PCNSTCL with isolated brain involvement occurred in two of 1,040 patients (0.19%). Out of 11 patients with CNS and systemic disease at presentation, brain involvement was documented in four patients, isolated leptomeningeal disease was diagnosed in four patients, combined parenchymal and leptomeningeal disease was detected in two cases, and leptomeningeal and ocular involvement was diagnosed in one patient (**Table 1**). Secondary CNS disease at relapse was documented as isolated brain involvement in five patients, as isolated leptomeningeal disease in six patients, and as combined parenchymal and leptomeningeal disease in five cases (**Table 2**).

### Prophylaxis and Treatment

Out of 1,027 patients without initial CNS disease, 579 received the first-line chemotherapy: cyclophosphamide, doxorubicin, vincristine, prednisone (CHOP); 185 received CHOP with etoposide (CHOEP); and 5 received fractionated cyclophosphamide, vincristine, doxorubicin, and dexamethasone alternating with high-dose methotrexate–cytarabine (Hyper-CVAD/MTX-AraC) (**Table 3**). CNS relapses occured in 9 (1.6%) of 579 patients treated with CHOP and in 7 (3.8%) of 185 patients treated with CHOEP.

**Table 3:** First-line treatment in patients with peripheral T-cell lymphomas without initial CNS involvement (*n* = 1,027).

Chemotherapy	Patients with subsequent secondary CNS relapse (*n* = 16 pts)	PTCL without CNS involvement (*n* = 1,011 pts)
CHOP	9	570
CHOEP	7	178
Hyper-CVAD/MTX+Cytarabine	0	5
Other	0	258

CHOP, cyclophosphamide, doxorubicin, vincristine, prednisone; CHOEP, cyclophosphamide, doxorubicin, vincristine, etoposide, prednisone; Hyper-CVAD, cyclophosphamide, vincristine, doxorubicin, dexamethasone; MTX, methotrexate.

Intravenous MTX 1 g/m^2^ and a high dose of AraC as part of complex hyper-CVAD/MTX-AraC chemotherapy were used in only five of 1,027 patients, and none of these five patients relapsed in CNS. Patients with testicular involvement (4 of 1,027 PTCL) and without CNS involvement received CNS prophylaxis with intrathecal MTX concomitantly with CHOP; otherwise, CNS prophylaxis with intrathecal MTX was not routinely administered. Despite intrathecal MTX prophylaxis, one of these 4 patients subsequently relapsed in leptomeninges.

Patients with PCNSTCL received MTX-based treatment (MTX/AraC/thiotepa in one patient and MTX-AraC in one patient) followed by high-dose chemotherapy (1× thiotepa and carmustine, 1× thiotepa, etoposide, AraC, and melphalan-TEAM) and ASCT (**Table 4**).

**Table 4:** Treatment of patients with peripheral T-cell lymphomas and CNS involvement at presentation or relapse (*n* = 29).

Treatment	Initial CNS disease (*n* = 13 pts)	Secondary CNS relapse (*n* = 16 pts)
**HD MTX based**	4	8
alone	0	1
+cytarabine	1	4
+cytarabine + thiotepa	1	0
+cytarabine + thiotepa + ICE	1	2
+IVE	1	0
+VCR, procarbazine, cytarabine	0	1
**Hyper-CVAD/MTX + cytarabine**	1	0
**MACOP B**	1	0
**IVAC**	1	0
**C(H)OP + intrathecal MTX**	6	0
**ICE**	0	3
**DHAP/DHAOx**	0	2
**Brentuximab vedotin**	0	1
**Gemcitabine/dexamethasone**	0	1
**Consolidation therapy**		
Autologous SCT	3	5
Allogeneic SCT	1	1
WBRT	2	5

HD MTX, high-dose methotrexate; ICE, ifosfamide, carboplatin, etoposide; IVE, ifosfamide, epirubicin, etoposide; Hyper-CVAD, cyclophosphamide, vincristine, doxorubicin, dexamethasone; MACOP B, methotrexate, doxorubicin, cyclophosphamide, vincristine, prednisone, bleomycin; IVAC, ifosfamide, etoposide, high-dose cytarabine; CHOP, cyclophosphamide, doxorubicin, vincristine, prednisone; DHAP, dexamethasone, cytarabine, cisplatin; DHAOx, dexamethasone, cytarabine, oxaliplatin; SCT, stem cell transplantation; WBRT, whole brain radiotherapy.

One young patient with CNS and extensive systemic involvement at diagnosis received a high dose of MTX in combination with AraC and thiotepa. As the patient did not achieve a complete remission, subsequent treatment included ifosfamide, carboplatin, etoposide (ICE), ASCT, and allogeneic stem cell transplantation. Heterogeneous treatment approaches were used in another four patients with initial CNS and systemic involvement (high dose of MTX followed by ifosfamide, epirubicin, and etoposide in one patient; Hyper-CVAD/MTX-AraC in one patient; methotrexate, doxorubicin, cyclophosphamide, vincristine, prednisone, and bleomycin-MACOP-B in one patient; and ifosfamide, etoposide, and AraC-IVAC in one patient) (**Table 4**). None of these 4 patients received consolidation treatment. Six patients with initial CNS and systemic involvement not eligible for a high dose of MTX received CHOP and intrathecal MTX. Consolidation whole brain radiotherapy (WBRT) was indicated in two of them (**Table 4**).

Eight patients with secondary CNS disease at relapse received high-dose MTX-based treatment. Additionally, intrathecal MTX was administered in six patients with leptomeningeal involvement (**Table 4**). Other treatment options at CNS relapse included the following: ifosfamide, carboplatin, and etoposide (ICE) in three patients; cytarabine, cisplatin, and dexamethasone (DHAP) in one patient; and dexamethasone, oxaliplatin, and cytarabine (DHAOx) in one patient. Brentuximab vedotin, an anti-CD30 antibody, anti-tubulin drug conjugate was used in a patient with relapsed CD30-positive ALCL with systemic and CNS disease after previous MTX failure. Brentuximab vedotin exhibits poor CNS penetration, and it was used in combination with gemcitabine and dexamethasone (27) (**Table 4**). ASCT was used in five patients after application of agents able to achieve therapeutic concentrations in the CNS tissue (2× thiotepa and carmustine, 2× BEAM, and 1× TEAM), and one of these patients subsequently underwent allogeneic stem cell transplantation (**Table 4**). Out of five cases receiving WBRT at CNS relapse, two received ASCT as well (**Table 4**).

### Survival and Prognostic Factors

**Figure 1:**
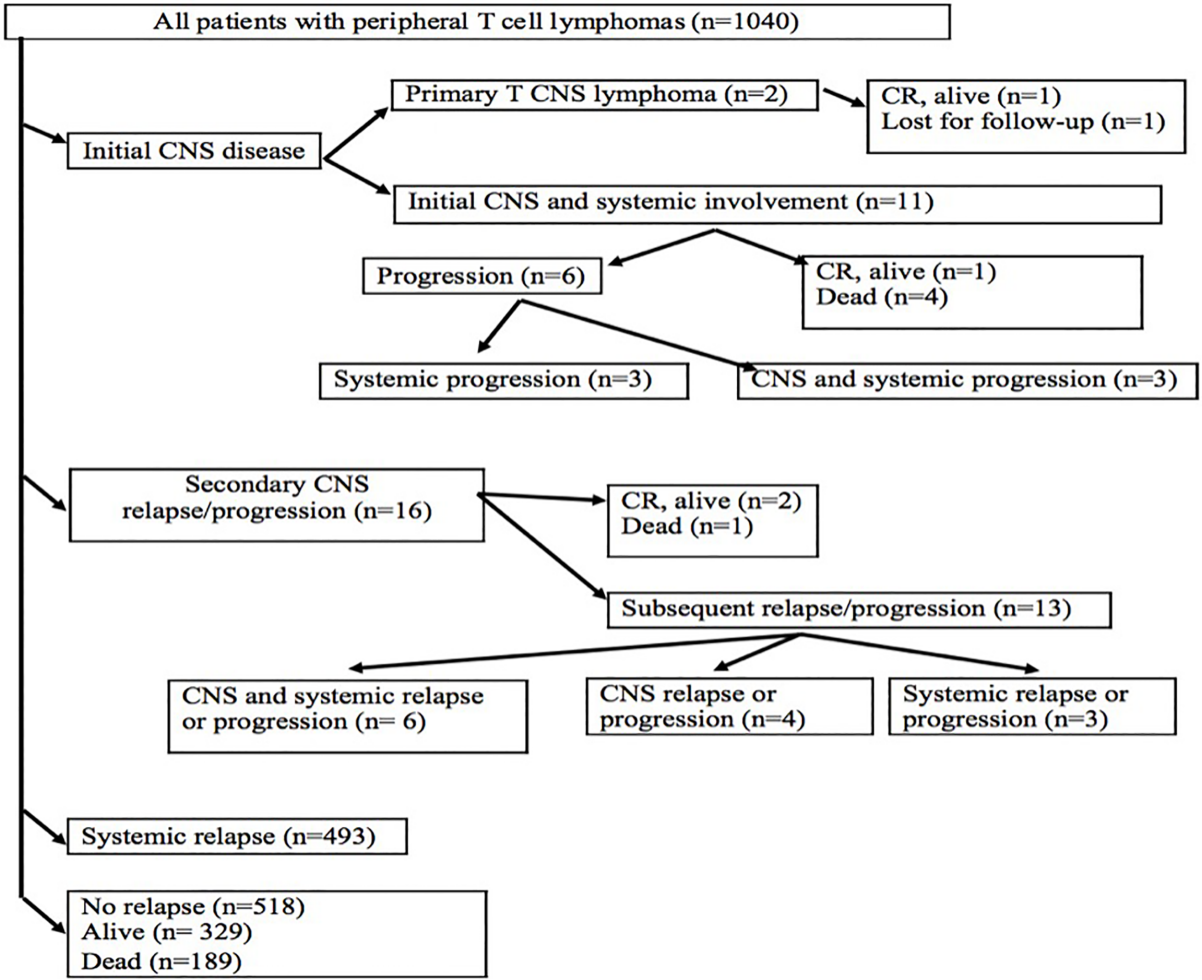
Flowchart of patients with peripheral T-cell lymphomas. CNS, central nervous system; CR, complete remission.

The median follow-up of 1,040 patients was 25.8 months and that of living patients with PTCL was 71.3 months. Two patients with PCNSTCL achieved a complete remission after the first-line treatment, and one of them remained alive; the second patient is lost to follow-up (**Figure 1**). Out of 11 cases with initial CNS and systemic disease after the first-line treatment, one patient treated with the most aggressive therapy (high dose of MTX, AraC, thiotepa, ICE, ASCT, and allogeneic stem cell transplantation) is alive in CR. Despite treatment with CHOP and intrathecal MTX, six patients progressed (**Figure 1**). Progressions included systemic disease, and persistent CNS disease was identified in three of them. The remaining four patients died due to treatment toxicity (2 due to sepsis, 1 due to febrile neutropenia of unknown origin, and 1 due to acute liver failure) without evaluation of disease status.

Secondary CNS relapses occured in 16 patients: at the first relapse in 13 patients, at the second relapse in two patients, and at the fourth relapse in one patient (**Figure 1**). Risk factors for CNS relapse included the following: involvement of more than one extranodal site (*p* = 0.008, HR = 0.96), soft tissue involvement (*p* = 0.003, HR = 6.3), testicular involvement (*p* = 0.046, HR = 1.58), and the presence of B symptoms (*p* = 0.035, HR = 0.91) (**Table 5**). Other factors like age, gender, clinical stage, ECOG performance status, bulky mass ≥10 cm, elevated LDH, IPI, and involvement of bone marrow, gastrointestinal tract, and skin were not statistically significant for a new CNS relapse (**Table 5**).

**Table 5:** Risk factors for secondary CNS relapse in peripheral T-cell lymphomas.

Risk factors	Secondary CNS relapse (*n* = 16 pts)	PTCL without CNS involvement (*n* = 1,011 pts)	*p*
**B symptoms**			
Yes	12	495	0.035
No	4	516	
**Extranodal site**			
0–1	8	811	
2	8	200	0.008
**Soft tissue involvement**			
Yes	4	39	0.003
No	12	972	
**Testicular involvement**			
Yes	1	3	0.046
No	15	1008	
**Bone marrow involvement**			
Yes	3	191	0.988
No	13	820	
**Gastrointestinal involvement**			
Yes	3	103	0.309
No	13	908	
**Paaranasal sinuses**			
Yes	1	10	0.159
No	15	1,001	
**Lung involvement**			
Yes	3	99	0.282
No	13	912	
**Pleural involvement**			
Yes	0	20	0.426
No	16	991	
**Bone involvement**			
Yes	2	44	0.194
No	14	967	
**Skin involvement**			
Yes	4	215	0.723
No	12	796	
**Kidney involvement**			
Yes	1	6	0.093
No	15	1,005	

**Figure 2:**
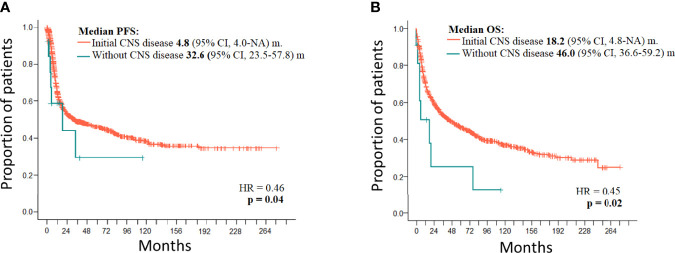
**(A)** PFS of PTCL with initial CNS disease (*n* = 11) and without initial CNS disease (*n* = 1,027). **(B)** OS of PTCL with initial CNS disease (*n* = 11) and without initial CNS disease (*n* = 1,027).

**Figure 3:**
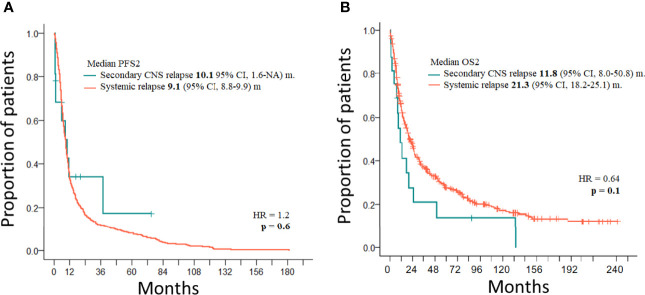
**(A)** PFS2 of PTCL with secondary CNS disease (*n* = 16) and with systemic relapse (*n* = 493). **(B)** OS2 of PTCL with secondary CNS disease (*n* = 16) and with systemic relapse (*n* = 493).

Treatment of 16 secondary CNS relapses resulted in two complete remissions (one after allogeneic stem cell transplantation and one after ASCT and WBRT), one death due to treatment related toxicity, and 13 relapses/progressions (**Figure 1**). Out of 27 patients with secondary CNS disease (excluding 2 cases of PCNSTCL), three patients (11.1%) are alive and 24 patients died. Causes of death included the following: 13 (54.2%) progressions/relapses of PTCL, 6 (25.0%) infections, 3 (12.5%) secondary malignancies—2 myelodysplastic syndromes occurring 14 and 20 months after diagnosis of PTCL and 1 Hodgkin lymphoma occurring 115 months after initial diagnosis, 1 (4.1%) CNS hemorrhage, and 1 (4.1%) acute liver failure. Systemic relapses/progressions were detected in 493 patients with PTCL. Out of 593 patients without a relapse, 329 patients are alive. The median PFS and OS of 1,038 patients (excluding PCNSTCL) were 32.4 months (95% CI 23.2–54.0) and 45.4 (95% CI 35.3–58.6) months, respectively. The median PFS of 1,027 patients (without initial CNS disease) was 32.6 months (95% CI 23.5–57.8) and the median PFS of 11 patients with initial CNS and systemic disease was 4.8 months (95% CI 4.0–NA); the difference was significant (*p* = 0.04, HR = 0.46) (**Figure 2A**). The median OS of 1,027 patients without initial CNS disease was 46.0 months (95% CI 36.6–59.2), and the median OS of 11 patients with initial CNS and systemic disease was 18.2 months (95% CI 4.8–NA); the difference was significant (*p* = 0.02, HR = 0.45) (**Figure 2B**). The median PFS of 1,011 patients without a CNS disease was 35.9 months (95% CI 24.1–64.2). The median PFS2 of 493 patients with relapse outside of CNS was 9.1 months (95% CI 8.1–9.9) and the median PFS2 of 16 patients with secondary CNS disease was 10.1 months (95% CI 1.6–NA) (**Figure 3A**). The difference was not significant (*p* = 0.6, HR = 1.2). The median OS of 493 patients with relapse outside of CNS was 21.3 months (95% CI 18.2–25.1) and the median OS of 16 patients with secondary CNS disease was 11.8 months (95% CI, 8.0–50.7) (**Figure 3B**). CNS involvement was not associated with a significantly worse OS compared with relapsed/refractory patients without CNS involvement (*p* = 0.1, HR = 0.64). The median OS of patients without a CNS relapse was 47.4 months (95% CI 37.5–62.6).

## Discussion

The overall incidence of CNS involvement in PTCL from NiHiL at initial diagnosis (1.25%) and at relapse (1.54%) was lower than reported in the literature (4, 6, 8, 11). Our data could be underestimated because CNS-directed staging and restaging examinations are not routinely performed in patients with T-cell lymphomas unless neurologic symptomatology is present and patients could die before the detection of the CNS event. We identified two PCNSTCLs at presentation with isolated brain involvement, and this rare event is in concordance with the reported data (22). The incidence of PCNSTCL is significantly lower than PCNSL in B-cell lymphomas (2% of all brain tumors and 5%–8% of extranodal lymphomas). Our data confirm the findings of published reports that systemic involvement concurrently with CNS disease is documented in majority of patients at presentation as well as at relapse, and the most common histology is PTCL NOS. Both concomitant CNS and systemic disease and histologic subtypes correspond with the Korean series in relapsed PTCL where CNS recurrence has been associated with systemic relapse in 90% of patients (28). Our data are also similar with European case series in CNS relapses (8). On the contrary, the US data showed that leptomeninges were the exclusive site of CNS relapse in all cases (10).

We identified the following risk factors for CNS relapse in our PTCL patients without initial CNS disease: involvement of more than one extranodal site, soft tissue involvement, testicular involvement, and the presence of B symptoms (**Table 5**). Involvement of >1 extranodal site was reported by several authors as a risk factor for CNS relapse in PTCL (8, 10, 11, 19). B symptoms and involvement of two or more extranodal organs are included as risk factors in CNS-PINK score for ENKTCL treated with non-anthracycline-based regimens (19). The risk of CNS relapse is 15% in primary testicular lymphomas of B-cell origin (29); however, the incidence of testicular involvement in PTCL is rare (8), and our observation of testicular involvement as a risk factor should be confirmed by further studies.

Several retrospective analyses demonstrated that intrathecal prophylaxis is not associated with reduced risk of CNS recurrence and a high incidence of leptomeningeal relapses was observed (8, 10, 11). No definitive conclusion can be drawn on the prophylactic effect of MTX on CNS relapses in our cohort due to a low number of patients treated this way; however, a subset of patients could potentially benefit from CNS prophylaxis as four patients with testicular disease received prophylactic intrathecal MTX concomitantly with CHOP and only one patient subsequently relapsed in leptomeninges. Hyper-CVAD/MTX-AraC chemotherapy was used in five of 1,027 patients, and none of these five patients relapsed in CNS. Despite a good CNS bioavailability of etoposide, our data do not support its prophylactic effect on the reduction of CNS relapse risk: CNS relapses occured in 3.8% of patients after CHOEP chemotherapy and in 2.6% of cases treated with CHOP. Etoposide did not reduce the CNS relapse rate also in the analysis from the Swedish Lymphoma Registry (8).

Heterogeneous treatment regimens of CNS involvement in our group of patients reflect the fact that there is no standard effective treatment of CNS involvement in PTCL. All retrospective studies reported high mortality rates. Overall, 26 cases had concomitant CNS and systemic involvement in our analysis. High or intermediate doses of MTX alone or in various combinations were used in 44.8% of our patients with CNS involvement either at presentation or at relapse; other patients were unable to receive MTX due to the age or comorbidities (**Table 4**).

Following the recommendations in aggressive B-cell lymphomas, overall eight patients received consolidation with ASCT (23–26) including two patients with PCNSTCL. Patients who are transplanted in a complete remission have better outcome, but it is possible that patients in partial remission could also benefit from ASCT (20, 22). According to the current approach in aggressive B-cell lymphomas, consolidation with WBRT is recommended in patients ineligible for ASCT, unable to collect stem cells, or not achieving a remission after induction. WBRT is associated with neurologic toxicity. Allogeneic stem cell transplantation was indicated in two patients; however, this approach remains controversial.

The median PFS of 11 patients with initial CNS involvement was very short (4.8 months), and only one patient remained in CR after extensive treatment (**Figure 2A**). The median OS of patients with initial CNS involvement is significantly worse when compared to patients without initial CNS involvement (*p* = 0.02, HR = 0.45) (**Figure 2B**). The fact that all relapses included disease outside of CNS and three of the six cases also had CNS disease indicates that neither MTX nor concomitant systemic chemotherapies are sufficient to control systemic and CNS disease in PTCL. A similar observation is true for the treatment of 16 secondary CNS relapses where the median PFS2 after salvage treatment was 10.1 months. The difference between median PFS2 and OS2 in CNS and systemic relapse was not significant, and our observation is in concordance with other studies demonstrating the overall poor prognosis of relapsed/refractory PTCL (8, 10, 11, 15, 28). Published data reported median survival shorter than 6 months, and only 10% of patients remained alive at 1 year from CNS relapse (8, 10, 11, 15). Out of all patients with secondary CNS lymphomas, only three (11.1%) patients in our study are alive and 24 patients died. The most common cause of death was progression/relapse of PTCL either systemic or in CNS. Not surprisingly, high mortality risk was associated with treatment-related infections (20.2%). Retrospective data analysis and the low number of patients with CNS disease at diagnosis or at relapse are the major limitations of our study.

In conclusion, the incidence of CNS disease at the time of diagnosis and at relapse in PTCL is low and usually associated with other systemic involvement. The prognosis of PTCL with initial CNS involvement is significantly worse when compared to patients without CNS disease at presentation. The outcome of CNS relapse is comparable with relapsed PTCL outside of CNS. The optimal treatment is not defined yet.

## Data Availability Statement

The original contributions presented in the study are included in the article/supplementary material. Further inquiries can be directed to the corresponding author.

## Ethics Statement

The studies involving human participants were reviewed and approved by the Ethics Committee of University Hospital Kralovske Vinohrady, Prague, Czechia. The patients/participants provided their written informed consent to participate in this study.

## Author Contributions

All authors listed have made a substantial, direct, and intellectual contribution to the work and approved it for publication.

## Funding

This work was supported by the Research project Cooperatio which was awarded by the Third Faculty of Medicine of Charles University in Prague, the Czech Republic.

## Conflict of Interest

The authors declare that the research was conducted in the absence of any commercial or financial relationships that could be construed as a potential conflict of interest.

## Publisher’s Note

All claims expressed in this article are solely those of the authors and do not necessarily represent those of their affiliated organizations, or those of the publisher, the editors and the reviewers. Any product that may be evaluated in this article, or claim that may be made by its manufacturer, is not guaranteed or endorsed by the publisher.
